# Health Care Utilization by Children With Asthma

**Published:** 2008-12-15

**Authors:** Hyoshin Kim, Gail M. Kieckhefer, Jutta M. Joesch, April A. Greek, Nazli Baydar

**Affiliations:** Battelle Centers for Public Health Research and Evaluation; University of Washington, Seattle, Washington; University of Washington, Seattle, Washington; Battelle Centers for Public Health Research and Evaluation, Seattle, Washington; Department of Sociology, Koç University, Istanbul, Turkey

## Abstract

**Introduction:**

We examined how differences in health service utilization among children with asthma are associated with race/ethnicity, socioeconomic status (family income, mother's education), and health insurance coverage.

**Methods:**

We analyzed Medical Expenditure Panel Survey data from 1996 through 2000 (982 children younger than 18 years with asthma). We calculated percentages and mean distributions, odds ratios, and incidence rate ratios.

**Results:**

Non-Hispanic black children used more urgent care services and fewer preventive health services. Children in low-income families (125%-199% of the poverty line) had the lowest levels of prescription fills and general checkups. Children whose mothers had more education had more checkups and fewer emergency department visits. Children who were insured during the 2-year study period used more health services for asthma, not including emergency department visits.

**Conclusion:**

Minority children and children of socioeconomically disadvantaged families use more urgent care and less preventive care for asthma. Children without health insurance use fewer health services overall. Future research should address how related factors might explain health services utilization in effectively managing asthma in children.

## Introduction

The prevalence of asthma, one of the most common chronic childhood illnesses in the United States, has steadily increased among children in the last 2 decades (1-3) and was 8.9% in 2005 (4). Studies have reported significantly higher rates of asthma prevalence, hospitalization, and death in minority children than in white children (5-14), and studies of Medicaid records show that black and Hispanic children have more emergency department visits for asthma and use fewer preventive asthma medications than do white children (7-11,15). Because childhood asthma results in a large number of physician visits, hospital admissions, and school absences (4,16-20), it disproportionately affects economically disadvantaged families (21-23).

Studies of asthma hospitalization and emergency department visits have concluded that ethnic disparities persist, even after adjusting for socioeconomic status (SES) (10,12,15); however, many past studies used limited samples, such as children on Medicaid (7-11,15), so results are not generalizable to all children or to children who lack health insurance. Furthermore, few studies have considered categories of health service utilization other than emergency department visits or hospitalization (10-13,15,24). Because most children with asthma do not use the emergency department or require hospitalization, inferences from such studies afford a limited view of the overall use of health services for asthma.

The goal of our study was to examine the extent to which race/ethnicity, SES, and health insurance status are associated with differences in utilization of 4 categories of health services (office visits, emergency department visits, prescribed medications, and general checkups) by children with asthma. A better understanding of the factors associated with disparities in utilization of health services for asthma may help policy makers develop health policies that can reduce these disparities.

## Methods

### Data source

The Medical Expenditure Panel Survey (MEPS) is an ongoing, nationally representative, in-home survey designed to provide information about health services use, expenditures, and insurance coverage of the US civilian, noninstitutionalized population (25,26). Conducted jointly by the Agency for Healthcare Research and Quality and the National Center for Health Statistics, MEPS uses an overlapping panel design in which a new cohort is initiated at the beginning of each year. The sampling frame for MEPS is drawn from respondents to the previous year's National Health Interview Survey and oversamples Hispanic and black households.

All information on a child's health and health service utilization, with the exception of pharmacy records, was collected by interviewing the adult household member who was most knowledgeable about the family's health care and who kept records about health service utilization (usually the mother). Individual records of prescribed medicines were collected from pharmacy records (25,26).

During the MEPS interview, respondents are asked, for each child in the household, 1) whether any medical conditions bothered the child during the period covered by the interview and 2) whether the child had medical conditions that resulted in missed school days, days spent in bed, or medical events (eg, health care visit or prescription fill) during the period covered by the interview. Therefore, MEPS identifies children as having asthma when the respondent reports either that the child was bothered by asthma or that asthma was the reason for a school absence, bed day, medical visit, or prescription fill during the period covered by the interview.

We pooled data from the initial 4 MEPS panels from 1996 through 2000. Because we focused on health service utilization during 2 calendar years in the panel, children who first reported asthma in the middle of a panel period were excluded from the analyses. We thus excluded 329 children, for a final sample of 982 children who were younger than 18 years at the start of each panel, had asthma at the beginning of the panel, and had valid response data. The only significant demographic differences between the children who were included and those who were excluded were in age and residence in a metropolitan area.

### Measures

Outcome measures in this study — health service utilization associated with asthma during 2 calendar years — were constructed for 4 categories of health services: 1) office and outpatient visits (hereafter referred to as office visits), 2) emergency department visits, 3) prescription fills from the reporting pharmacy, and 4) well-child care and general checkups (hereafter referred to as checkups). Information on the use of inpatient care was available, but because of infrequent use, this category was not analyzed. Total number of checkups was calculated by summing office visits for general checkup, vaccination, or well-child examination, regardless of asthma condition. If office visits were made for emergency reasons, those visits were included in the emergency department category rather than in the office visit category. For these 4 categories of health services, we constructed an indicator of whether the child ever used that service because of asthma. The number of such services used was summed for the 2 calendar years.

We included race/ethnicity, SES, health insurance status, and other control variables likely to be associated with asthma-related health service utilization in the multivariate models. Race/ethnicity was included in the models as Hispanic, non-Hispanic black, and non-Hispanic white and other racial groups of children (hereafter referred to as Hispanic, black, and white, respectively). Other races were combined with the white group because of the small number of children in other racial groups (n = 22: 3 American Indian, 16 Asian or Pacific Islander, and 3 "other"). We also conducted analyses that excluded these 22 children, and the results were essentially the same.

Our 2 measures of SES were annual family income and mother's education. MEPS provides a categorical variable for annual family income as a percentage of the federal poverty line by using the following 5 categories: less than 100% (*poor*), 100% to 124% (*near poor*), 125% to 199% (*low income*), 200% to 399% (*middle income*), and 400% or more (*high income*) of the poverty line. For family income, we used annual income; if the family's annual income differed between the 2 years of the study, we used the lower number. Mother's education was included in the models because mothers with higher education were expected to understand that regular use of health services might prevent asthma flare-ups (27).

Children's health insurance coverage was also included because it is likely to influence how parents use health services for asthma. Measures of health insurance coverage were constructed by combining yearly insurance coverage variables provided by MEPS. We assigned those children who were uninsured for an entire year to the category of *uninsured*, those who had public insurance coverage for both years to *public*, and the rest to *private/public*. We also conducted analyses that separated *private* only from *private/public*, and the results were essentially the same.

Other control variables such as age and sex were also included in the model. Family size was dichotomized as less than 5 and 5 or more. Indicators of region as well as metropolitan statistical area residence were included to control for regional and urban variability in access to health care. Indicators for different panel periods were included to control for systematic factors during the survey periods, such as changes in asthma management guidelines at the national level, and for any variability in health insurance policies.

### Data analysis

We performed descriptive analyses to examine demographic characteristics and distribution of health service utilization. Multivariate analyses examined disparities in health service utilization predicted by major sociodemographic and health insurance variables while controlling for other background characteristics. For the multivariate analyses, we used logistic regression to estimate odds ratios (ORs) and negative binomial regressions to estimate incidence rate ratios (IRRs). The 2 estimates represent the use of 2 different types of measures for health service utilization: the first is whether or not children with asthma ever use a certain health service, and the second is how often they use the service.

In estimating ORs by using logistic regression, we used dichotomous dependent variables for whether children used each category of health service in 4 separate models. We dichotomized race/ethnicity, family income, mother's education, health insurance coverage, and other demographic characteristics to use as predictors in the models. To estimate IRRs using negative binomial regressions, data for the number of times each health service was used were employed in 4 separate models (28). The same set of predictors included in the logistic regressions was used to estimate IRRs in negative binomial regressions. All estimates were weighted to account for the complex sampling design of MEPS, thereby providing nationally representative figures. Estimates were considered significant at *P* ≤ .05, but we examined the magnitude and direction of estimates regardless of significance.

## Results

Approximately one-fourth of children with asthma were black, and approximately one-third lived below the federal poverty line (Table 1). Asthma was less common in girls and in children who were younger than 5 years.

Approximately 7.5% of children with asthma used no health services during the 2-year study period. The most frequently used type of health service was prescription fills, followed by general checkups and office visits (Table 2). Approximately 14% had an asthma-related emergency department visit during the study period.

Distribution of health service utilization varied significantly by family characteristics (Table 2). Black children had the lowest level of office visits and the highest level of emergency department utilization. Children in low-income families had the lowest level of prescription fills. Children with mothers who had the least education had the lowest level of prescription fills and checkups. Uninsured children had the lowest level of health service utilization across all categories except for office visits.

During the study period, black children and children whose mothers had less than a high school education used health services the fewest number of times, except for the emergency department, which they used the most number of times (Table 3). Because few children used the emergency department, the average number of visits was small. Of those who used the emergency department, 70% used it only once, and the maximum number of visits was 3. Overall, black children, children from low-income families, children whose mothers had less than a high school education, and children who had no insurance had the fewest checkups.

Minority children were more likely to use the emergency department for asthma than were white children (Table 4). Mothers who had the most education were most likely to have their children's prescriptions filled and take their children for checkups. Children who had health insurance were more likely to have checkups than were uninsured children. Family income variables were not significantly associated with the odds of using health services, but the pattern suggests that children in poorer families (income <200% of the poverty line) were less likely to use health services than were children in middle- and high-income families. For control variables (data not shown), children aged 5 to 10 years and girls were less likely to have checkups than were older children and boys, respectively. Children who lived in regions other than the West were more than twice as likely to use the emergency department and were also more likely to have checkups.

IRRs shown in Table 5 confirm the pattern of OR results in Table 4, but they provide additional information about the extent of health service utilization. Black children had more emergency department visits and fewer checkups than did other children. Hispanic children had twice as many emergency department visits as did white children and more office visits than either black or white children. With respect to family income, children in low-income families had significantly fewer visits for checkups than did children in high-income families. A similar pattern was found for prescribed medications, although it did not reach significance (*P* = .07). Children whose mothers had more education visited the emergency department less often and had checkups more often. Children who had only public health insurance used more of all types of health services (although this difference did not reach significance for emergency department utilization).

Most control variables were not significantly associated with frequency of health service utilization, except that younger children had more emergency department visits and fewer prescription fills and checkups. Children who lived in regions other than the West had more health service utilization in general (data not shown).

## Discussion

We found that race/ethnicity, health insurance coverage, and family income and mother's education (as proxies for SES) are associated with differences in all categories of health service utilization for children with asthma. In general, minority children, poor children, children who lack health insurance, and children whose mothers are less educated use more emergency department care and less preventive care.

Consistent with past studies of race/ethnicity, emergency department utilization for asthma is significantly higher for black and Hispanic children than for white children, even after controlling for other socioeconomic factors (10). Part of the explanation may be that black and Hispanic (especially Puerto Rican) children have more asthma attacks than do whites (as supported by trend data [3,6]), but the explanation may also lie with the lack of a usual source of care, with level of home asthma management skills, or with attitudes (13,15). If parents rely on emergency department care for children who are having acute asthma episodes because they lack home management skills, then intervention programs designed to improve caregivers' asthma management skills or attitudes would be useful. However, if parents seek emergency department care for asthma episodes because they lack access to usual care, then more systems-oriented changes may be needed.

Our findings do not show significant differences in prescription filling by race/ethnicity. This finding is in contrast to those of previous studies of children on Medicaid (9,15), which reported that minority children are less likely to use antiinflammatory or controller medications to prevent asthma exacerbations. The discrepancies between these findings may come from differences in the samples (national vs Medicaid). We do not report the analyses of antiinflammatory or controller medications alone because MEPS provides data only on which medications were prescribed, not which medications were used.

We found that Hispanic children have a significantly higher level of office visits than do children of other racial/ethnic groups. Puerto Ricans have a much higher prevalence of asthma than do other racial/ethnic groups or Hispanic subgroups (6), which may explain why Hispanic children have more office visits for asthma than do white or black children. We tested this explanation in a separate analysis by including Puerto Ricans as a separate ethnic group, but we found that non-Puerto Rican Hispanic groups had significantly more office visits than did other groups (data not shown). The small sample sizes of other Hispanic subgroups prohibited further investigation of subgroup differences.

The associations with family income are less straightforward, and most of the associations in multivariate analysis did not reach significance. Only children in low-income families (125%-199% of the poverty level) had significantly fewer checkups. Nevertheless, the overall pattern suggests that children from families at less than 200% of the poverty level use less preventive and urgent care than do children from high-income families. Low-income families may lack the resources needed to manage their children's asthma and prevent exacerbations.

Children with health insurance are more likely to use health services and use more services than children without insurance, although the association usually reached or approached significance in children with only public insurance. The reasons for this finding may be complex. Some children in low-income families who do not have private insurance are likely to be ineligible for public insurance. Consequently, they may not use health services as much as children of poor or near-poor families who have public insurance. In our sample, approximately 17% of children in low-income families were uninsured, compared with 9% in poor families and 10% in near-poor families. Approximately 65% of children in poor families and 40% in near-poor families had only public insurance, compared with 20% of children in low-income families. Public health insurance appears to provide support for poor families, which suggests that children with asthma in low-income families that lack insurance may be in the most vulnerable position.

Children whose mothers had the most education received more preventive care and had fewer emergency department visits than children whose mothers had less education. This finding reinforces the idea that mothers with more education focus more on prevention and rely less on urgent care (15).

A strength of our study is that we used nationally representative data from MEPS. MEPS provides extensive information on health care utilization during 2 complete calendar years and concurrent socioeconomic and health insurance information for children with asthma.

We conclude that minority children of socioeconomically disadvantaged families use more urgent care and less preventive care for asthma, and families without health insurance use fewer health services overall. Providing health insurance to children who do not have it may be crucial to managing asthma, especially for preventive services such as filling prescriptions and having general checkups. To meet the needs of more uninsured children, the federal government should support public insurance programs for underserved children, such as the State Children's Health Insurance Program. Future research should address how additional factors — such as caregiver time, skills, and attitudes — relate to health services utilization in children with asthma.

## Figures and Tables

**Table 1:** Demographic Characteristics of Children With Asthma in MEPS Panel 1 (1996-1997) Through Panel 4 (1999-2000) (N = 982)

**Variable**	**% (SE)^a^**
**Race/ethnicity**	
Hispanic	14.8 (0.5)
Non-Hispanic black	23.5 (1.2)
Non-Hispanic white (includes all others)	61.7 (2.3)
**Family income as a percentage of the poverty line, %**	
<100 (poor)	31.1 (1.4)
100-124 (near poor)	7.7 (2.0)
125-199 (low income)	18.6 (2.0)
200-399 (middle income)	28.1 (1.9)
≥400 (high income)	14.5 (1.6)
**Mother's education, y**	
≤11	18.0 (1.6)
12	37.5 (2.2)
≥13	38.0 (2.2)
Missing data	6.5 (1.1)
**Health insurance coverage**	
Private/public (includes private only)	62.8 (2.1)
Public only	27.7 (2.0)
Uninsured	9.5 (1.1)
**Age, y**	
≤4	21.8 (1.6)
5-10	38.1(1.9)
11-17	40.1 (1.9)
**Sex**	
Male	63.8 (1.9)
Female	36.2 (1.9)
**Family size**	
<5	60.9 (2.1)
≥5	39.1 (2.1)
**Region of residence**	
Northeast	20.3 (2.1)
Midwest	23.2 (2.4)
South	33.8 (2.4)
West	22.6 (2.0)
**Residence**	
In MSA	80.5 (1.9)
Not in MSA	19.5 (1.9)
**Panel**	
Panel 1 (1996-1997)	28.9 (2.0)
Panel 2 (1997-1998)	25.0 (2.3)
Panel 3 (1998-1999)	19.6 (1.8)
Panel 4 (1999-2000)	26.5 (2.4)

Abbreviations: MEPS, Medical Expenditure Panel Survey; SE, standard error; MSA, metropolitan statistical area.

a Adjusted for survey design effects. Percentages may not add to 100% because of rounding.

**Table 2:** Distribution of Children With Asthma Who Have Ever Used Each Type of Health Care Service for Asthma During 2 Calendar Years, MEPS, 1996-2000 (N = 982)

Variable	Type of Health Care Service^a^			

	Office^b^	ED	Prescription	Checkup^c^
**Race/ethnicity**				
Hispanic (n = 280)	64.2 (3.7)	16.2 (2.7)	82.8 (2.6)	59.8 (4.1)
Non-Hispanic black (n = 243)	50.4 (3.7)	24.9 (3.5)	80.9 (3.6)	61.0 (3.9)
Non-Hispanic white (includes all others) (n = 459)	58.8 (2.8)	9.2 (1.5)	82.7 (2.0)	65.1 (2.9)
*P* value	.05	<.001	.68	.46
**Family income as a percentage of the poverty line, %**				
<100 (poor) (n = 378)	51.6 (3.5)	19.1 (2.5)	79.0 (2.7)	60.7 (3.3)
100-124 (near poor) (n = 84)	63.9 (6.9)	14.1 (4.7)	76.7 (7.0)	59.3 (7.6)
125-199 (low income) (n = 170)	58.0 (4.5)	10.8 (2.8)	76.0 (4.0)	59.2 (5.2)
200-399 (middle income) (n = 241)	60.9 (3.7)	11.0 (2.2)	88.6 (2.0)	67.2 (3.5)
≥400 (high income) (n = 109)	60.3 (5.2)	12.7 (3.5)	86.8 (4.2)	69.1 (5.4)
*P* value	.32	.11	.03	.43
**Mother's education, y**				
≤11 (n = 336)	54.6 (4.6)	20.8 (3.6)	74.4 (3.3)	52.0 (4.5)
12 (n = 340)	59.5 (3.2)	11.6 (2.0)	82.2 (2.3)	65.7 (3.3)
≥13 (n = 237)	59.7 (3.1)	12.9 (2.0)	87.0 (2.3)	68.3 (3.2)
Missing data (n = 69)	43.0 (8.1)	14.5 (6.2)	73.9 (8.7)	52.2 (8.3)
*P* value	.21	.12	.02	.01
**Health insurance coverage**				
Private/public (includes private only) (n = 534)	59.1 (2.5)	11.2 (1.6)	84.4 (1.9)	65.1 (2.7)
Public only (n = 336)	54.6 (3.4)	21.6 (3.3)	79.8 (2.7)	66.6 (3.3)
Uninsured (n = 112)	56.2 (6.2)	9.8 (3.5)	73.7 (5.4)	42.1 (5.9)
*P* value	.54	.003	.06	<.001
**Total**	57.6 (1.9)	14.0 (1.4)	82.1 (1.7)	63.4 (2.1)

Abbreviations: MEPS, Medical Expenditure Panel Survey; ED, emergency department.

a All values are % (standard error) unless otherwise indicated. Values are adjusted for survey design effects. *P* values were calculated by using χ^2^ tests of independence.

b Office and outpatient visits.

c General checkup, vaccination, or well-child examination.

**Table 3:** Number of Times Selected Health Care Services Were Used by Children With Asthma During 2 Calendar Years, MEPS, 1996-2000 (N = 982)

Variable	Type of Health Care Service^a^			

	Office^b^	ED	Prescription	Checkup^c^
**Race/ethnicity**				
Hispanic (n = 280)	2.74 (0.51)	0.25 (0.06)	7.02 (0.85)	2.60 (0.56)
Non-Hispanic black (n = 243)	1.86 (0.28)	0.45 (0.07)	6.45 (0.78)	1.54 (0.20)
Non-Hispanic white (includes all others) (n = 459)	2.11 (0.23)	0.16 (0.04)	6.85 (0.62)	2.70 (0.33)
*P value*	.33	.004	.88	.01
**Family income as a percentage of the poverty line, %**				
<100 (poor) (n = 378)	1.83 (0.29)	0.35 (0.08)	6.33 (0.72)	2.10 (0.34)
100-124 (near poor) (n = 84)	3.80 (1.21)	0.31 (0.14)	7.28 (1.79)	3.80 (1.23)
125-199 (low income) (n = 170)	1.85 (0.28)	0.19 (0.06)	5.08 (0.69)	1.29 (0.19)
200-399 (middle income) (n = 241)	2.14 (0.26)	0.15 (0.04)	6.86 (0.73)	2.79 (0.50)
≥400 (high income) (n = 109)	2.34 (0.47)	0.20 (0.06)	9.50 (1.76)	3.08 (0.78)
*P* value	.40	.21	.11	.003
**Mother's education, y**				
≤11 (n = 336)	1.83 (0.27)	0.47 (0.15)	6.21 (0.82)	1.75 (0.38)
12 (n = 340)	2.16 (0.30)	0.16 (0.03)	6.72 (0.87)	2.22 (0.32)
≥13 (n = 237)	2.39 (0.30)	0.19 (0.04)	7.51 (0.78)	3.11 (0.47)
Missing data (n = 69)	1.54 (0.66)	0.34 (0.15)	4.48 (1.37)	1.32 (0.30)
*P* value	.50	.14	.28	.01
**Health insurance coverage**				
Private/public (includes private only) (n = 534)	2.14 (0.20)	0.19 (0.03)	6.99 (0.60)	2.56 (0.31)
Public only (n = 336)	2.32 (0.41)	0.38 (0.10)	7.04 (0.80)	2.49 (0.40)
Uninsured (n = 112)	1.67 (0.26)	0.16 (0.07)	4.66 (1.08)	1.22 (0.29)
*P* value	.26	.14	.13	.007
**Total**	2.14 (0.19)	0.24 (0.30)	6.78 (0.45)	2.41 (0.23)

Abbreviations: MEPS, Medical Expenditure Panel Survey; ED, emergency department.

a All values are mean (standard error) unless otherwise indicated. Values are adjusted for survey design effects. *P* values were calculated by using F tests for difference.

b Office and outpatient visits.

c General checkup, vaccination, or well-child examination.

**Table 4:** Use of Health Care Services by Children With Asthma, MEPS, 1996-2000 (N = 982)^a^

Variable	Type of Health Care Service							

	Office^b^		ED		Prescription		Checkup^c^	

	OR (95% CI)	*P*	OR (95% CI)	*P*	OR (95% CI)	*P*	OR (95% CI)	*P*
**Race/ethnicity**								
Hispanic	1.41 (0.85-2.32)	.18	1.86 (1.05-3.30)	.03	1.43 (0.81-2.50)	.22	1.04 (0.65-1.68)	.86
Non-Hispanic black	0.77 (0.51-1.15)	.20	2.96 (1.70-5.15)	<.001	0.90 (0.57-1.42)	.66	0.83 (0.52-1.31)	.42
Non-Hispanic white (includes all others)	1 [Reference]		1 [Reference]		1 [Reference]		1 [Reference]	
**Family income as a percentage of the poverty line, %**								
<100 (poor)	0.72 (0.41-1.29)	.27	0.72 (0.31-1.64)	.43	0.68 (0.29-1.61)	.37	0.73 (0.37-1.43)	.35
100-124 (near poor)	1.30 (0.60-2.84)	.50	0.65 (0.23-1.87)	.43	0.60 (0.22-1.63)	.32	0.87 (0.38-2.01)	.75
125-199 (low income)	0.97 (0.52-1.80)	.92	0.58 (0.22-1.58)	.29	0.56 (0.24-1.30)	.18	0.82 (0.40-1.66)	.56
200-399 (middle income)	1.06 (0.64-1.76)	.81	0.86 (0.43-1.75)	.69	1.30 (0.59-2.89)	.51	1.07 (0.60-1.91)	.83
≥400 (high income)	1 [Reference]		1 [Reference]		1 [Reference]		1 [Reference]	
**Mother's education, y**								
≤11	1 [Reference]		1 [Reference]		1 [Reference]		1 [Reference]	
12	1.17 (0.73-1.88)	.51	0.50 (0.27-0.95)	.03	1.51 (0.87-2.64)	.14	1.81 (1.07-3.06)	.03
≥13	1.20 (0.74-1.94)	.45	0.70 (0.39-1.26)	.24	2.07 (1.21-3.54)	.008	2.04 (1.22-3.42)	.007
Missing data	0.60 (0.27-1.35)	.22	0.58 (0.19-1.81)	.35	1.15 (0.49-2.67)	.75	0.99 (0.40-2.44)	.98
**Health insurance coverage**								
Private/public (includes private only)	0.99 (0.56-1.74)	.97	1.06 (0.45-2.54)	.89	1.28 (0.72-2.30)	.40	1.69 (0.98-2.91)	.06
Public only	1.13 (0.62-2.05)	.70	1.84 (0.75-4.51)	.18	1.63 (0.87-3.04)	.13	3.28 (1.73-6.24)	<.001
Uninsured	1 [Reference]		1 [Reference]		1 [Reference]		1 [Reference]	

Abbreviations: OR, odds ratio; CI, confidence interval; MEPS, Medical Expenditure Panel Survey; ED, emergency department.

a ORs and CIs are weighted. Control variables for multivariate analyses were age, sex, family size, residence in metropolitan statistical area, region of residence, and panel.

b Office and outpatient visits.

c General checkup, vaccination, or well-child examination.

**Table 5:** Number of Visits Made for Health Care Services During 2 Calendar Years by Children With Asthma, MEPS, 1996-2000 (N = 982)^a^

Variable	Type of Health Care Service							

	Office^b^		ED		Prescription		Checkup^c^	

	IRR (95% CI)	*P*	IRR (95% CI)	*P*	IRR (95% CI)	*P*	IRR (95% CI)	*P*
**Race/ethnicity**								
Hispanic	1.50 (1.03-2.20)	.04	2.02 (1.05-3.88)	.04	1.17 (0.89-1.53)	.26	1.12 (0.76-1.66)	.57
Non-Hispanic black	0.89 (0.62-1.28)	.53	3.34 (2.06-5.42)	<.001	0.97 (0.74-1.25)	.79	0.67 (0.49-0.91)	.01
Non-Hispanic white (includes all others)	1 [Reference]		1 [Reference]		1 [Reference]		1 [Reference]	
**Family income as a percentage of the poverty line, %**								
<100 (poor)	0.70 (0.45-1.11)	.13	0.88 (0.38-2.03)	.77	0.69 (0.44-1.09)	.11	0.72 (0.40-1.32)	.28
100-124 (near poor)	1.88 (0.95-3.72)	.07	0.79 (0.30-2.08)	.64	0.90 (0.51-1.56)	.70	1.26 (0.62-2.55)	.53
125-199 (low income)	0.91 (0.54-1.53)	.72	0.54 (0.22-1.36)	.19	0.65 (0.41-1.03)	.07	0.52 (0.31-0.86)	.01
200-399 (middle income)	0.98 (0.64-1.51)	.93	0.82 (0.41-1.63)	.57	0.78 (0.53-1.14)	.20	0.98 (0.57-1.69)	.94
≥400 (high income)	1 [Reference]		1 [Reference]		1 [Reference]		1 [Reference]	
**Mother's education, y**								
≤11	1 [Reference]		1 [Reference]		1 [Reference]		1 [Reference]	
12	1.16 (0.75-1.81)	.50	0.33 (0.17-0.65)	.001	1.05 (0.73-1.51)	.78	1.27 (0.83-1.94)	.26
≥13	1.43 (0.91-2.24)	.12	0.46 (0.24-0.87)	.02	1.20 (0.83-1.73)	.33	1.63 (1.02-2.59)	.04
Missing	0.64 (0.32-1.27)	.20	0.55 (0.21-1.45)	.23	0.73 (0.39-1.36)	.32	0.80 (0.46-1.40)	.43
**Health insurance coverage**								
Private/public (includes private only)	1.20 (0.85-1.71)	.29	1.33 (0.55-3.18)	.53	1.38 (0.93-2.05)	.11	1.51 (0.87-2.60)	.14
Public only	1.66 (1.11-2.50)	.02	1.66 (0.63-4.37)	.30	1.65 (1.13-2.40)	.009	2.07 (1.15-3.71)	.02
Uninsured	1 [Reference]		1 [Reference]		1 [Reference]		1 [Reference]	

Abbreviations: IRR, incidence rate ratio; CI, confidence interval; MEPS, Medical Expenditure Panel Survey; ED, emergency department.

a IRRs and CIs are weighted. Control variables for multivariate analyses were age, sex, family size, residence in metropolitan statistical area, region of residence, and panel.

b Office and outpatient visits.

c General checkup, vaccination, or well-child examination.
